# Resistin promotes tumor metastasis by down-regulation of miR-519d through the AMPK/p38 signaling pathway in human chondrosarcoma cells

**DOI:** 10.18632/oncotarget.2724

**Published:** 2014-11-06

**Authors:** Chun-Hao Tsai, Hsiao-Chi Tsai, Ho-Ning Huang, Chih-Hung Hung, Chin-Jung Hsu, Yi-Chin Fong, Horng-Chaung Hsu, Yuan-Li Huang, Chih-Hsin Tang

**Affiliations:** ^1^ Department of Medicine and Graduate Institute of Clinical Medical Science, China Medical University, Taichung, Taiwan; ^2^ Department of Orthopedic Surgery, China Medical University Hospital, Taichung, Taiwan; ^3^ Graduate Institute of Basic Medical Science, China Medical University, Taichung, Taiwan; ^4^ Department of Biotechnology, College of Health Science, Asia University, Taichung, Taiwan; ^5^ School of Chinese Medicine, College of Chinese Medicine, China Medical University, Taichung, Taiwan; ^6^ Department of Pharmacology, School of Medicine, China Medical University, Taichung, Taiwan

**Keywords:** Chondrosarcoma, MicroRNA, MMP, Resistin, Metastasis

## Abstract

Resistin is a recently discovered adipocyte-secreting adipokine, which may play a critical role in modulating cancer pathogenesis. Chondrosarcoma is a highly malignant tumor known to frequently metastasize; however, the role of resistin in the metastasis of human chondrosarcoma is largely unknown. Here, we found that the expression of resistin was higher in chondrosarcoma biopsy tissues than in normal cartilage. Moreover, treatment with resistin increased matrix metalloproteinase (MMP)-2 expression and promoted cell migration in human chondrosarcoma cells. Co-transfection with microRNA (miR)-519d mimic resulted in reversed resistin-mediated cell migration and MMP-2 expression. Additionally, AMP-activated protein kinase (AMPK) and p38 inhibitors or siRNAs reduced the resistin-increased cell migration and miR-519d suppression, and inhibition of resistin expression resulted in suppression of MMP-2 expression and lung metastasis *in vivo*. Taken together, our results indicate that resistin promotes chondrosarcoma metastasis and MMP-2 expression through activation of the AMPK/p38 signaling pathway and down-regulation of miR-519d expression. Therefore, resistin may represent a potential novel molecular therapeutic target in chondrosarcoma metastasis.

## INTRODUCTION

Chondrosarcoma is a cartilage forming neoplasm, and the second most common primary bone malignancy, representing approximately 40% of all primary bone cancers. Unlike other primary bone cancers, which mainly affect children and adolescents, chondrosarcoma can present at any age [1]. The development of distant metastases often leads to a significant decline in overall survival, regardless of tumor grade or localization [2]. Chondrosarcoma is resistant to both chemotherapy and radiation, and no specific standardized therapy has been developed for this malignancy, making wide local excision the only treatment option available [3]. Recently, many studies have focused on developing new targeted therapies, which include targeting the hedgehog pathway, inhibition of B-cell lymphoma-2 expression, and inhibition of inflammation responses, among others.

Resistin is a 12.5-kDa protein secreted from adipocytes and monocytes in humans, and which has been demonstrated to be involved in various inflammatory processes [4]. Recently, inflammation has been demonstrated to play a pathogenic role in cancer [5]. In addition, resistin expression has also been found to gradually increase along with the progression of certain tumors, including breast, prostate, colon, gastric, and endometrial cancers [6, 7]. Previous studies have demonstrated that resistin is involved in the metastasis of breast and lung cancers [8, 9]; however, the role of resistin in chondrosarcoma is currently largely unknown.

In most cancers, metastasis is a major clinical problem, and is responsible for approximately 90% of cancer patient mortality. During metastasis, through activation of specific signaling pathways, tumor cells secrete certain proteins, growth factors, and cytokines, allowing them to invade the surrounding tissues [10]. Matrix metalloproteinases (MMPs) are zinc-dependent endopeptidases and are considered as critical molecules assisting tumor metastasis [11]. In soft tissue sarcoma, clinical and experimental studies have demonstrated that elevated levels of MMPs are associated with tumor progression and shortened patient survival. MMP-1, MMP-2, MMP-3, MMP-9, and MMP-13 are expressed in human chondrosarcoma cells [12]. Among various MMP types, MMP-2 play pivotal roles in tumor cell invasion and metastasis by degradation of type IV collagen, the major component of the cartilage [13, 14]. It has been reported that MMP-2 inhibition suppressed migration and metastasis of chondrosarcoma [12, 15, 16]. Therefore, MMP-2 may a key regulator during metastasis of chondrosarcoma. AMP-activated protein kinase (AMPK) is a cellular energy sensor responsible for maintaining energy homeostasis. AMPK expression has been found to correlate with various cancers, including ovarian, hepatocellular, pancreatic, breast, and gallbladder cancers [17-22]. Moreover, several studies have also shown that AMPK plays an important role in cancer metastasis [17]. AMPK activation has been reported to mediate chemokine (C-C motif) ligand 3-increased MMP-2 expression and chondrosarcoma metastasis [23]. However, the effects of AMPK activation on resistin-mediated metastasis and MMP expression in human chondrosarcoma are currently unknown.

microRNAs (miRNAs) are small, endogenous, evolutionarily conserved non-coding ribonucleotide acids. It is estimated that up to 3% of the human genome codes for miRNA sequences [24, 25]. MiRNAs are involved in numerous biological processes, including cell growth, development, differentiation, proliferation, and death. They bind to complementary sequences in the 3′ untranslated regions (3′ UTRs) of their target mRNAs, resulting in degradation or blocking of gene translation [26]. Previously studies have demonstrated a role of miRNAs in modulating the metastatic process in many tumors [27]. MiR-519d has been considered as an onco-miRNA in tumor progression, which could up-regulated by p53 and DNA hypo-methylation and then target to CDKN1A/p21, PTEN, AKT3 and TIMP2 [28]. In addition, miR-519d regulating MMP-dependent migration and metastasis is documented [29]. In this study, whether miR-519d play a role in resistin-mediated metastasis was examined and found that resistin promotes tumor metastasis and MMP-2 expression by down-regulation of miR-519d expression through the AMPK/p38 signaling pathway in human chondrosarcoma.

## RESULTS

### Resistin promotes cell migration and MMP-2 expression in chondrosarcoma cells

**Figure 1:**
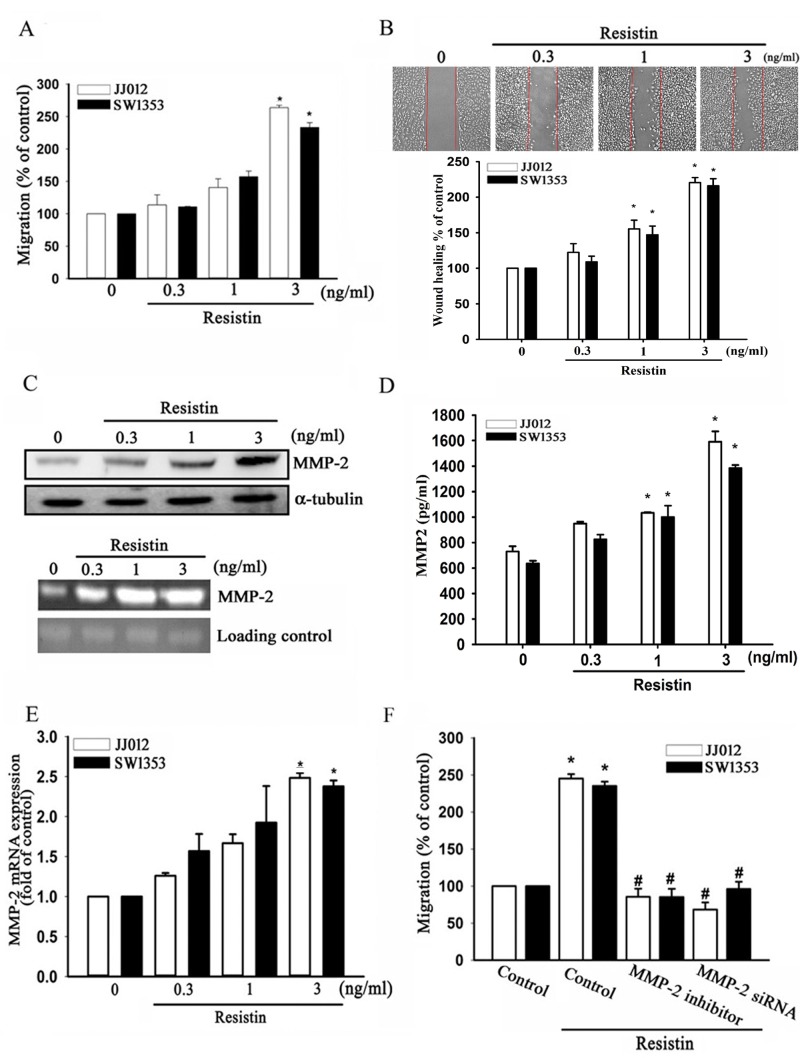
Resistin promotes cell migration of chondrosarcoma cells through increasing matrix metalloproteinase (MMP-2) expression The JJ012 and SW1353 cells were incubated with resistin (0.3–3 ng/ml) for 24 h, and *in vitro* migration was measured by (A) Transwell and (B) wound-healing assays. (C) JJ012 cells were incubated with resistin for 24 h, the protein and mRNA expressions of MMP-2 were measured by western blot (upper panel) and zymography (lower panel). The JJ012 and SW1353 cells were incubated with resistin for 24 h, and the protein and mRNA expressions of MMP-2 were measured by (D) enzyme-linked immunosorbent assay (ELISA), and (E) real-time quantitative polymerase chain reaction (RT-qPCR). (F) The JJ012 and SW1353 cells were pre-treated with an MMP-2 inhibitor or pre-transfected with MMP-2 siRNA, and the *in vitro* migration was measured using Transwell assays. The results are expressed as mean ± SEM. *, *P* < 0.05 compared with control. ^#^, *P* < 0.05 compared with the resistin-treated control group.

Distant metastasis of chondrosarcoma is associated with a poor prognosis and high mortality rate [30]. To investigate the effects of resistin on chondrosarcoma cell migration, JJ012 and SW1353 cells were treated with different concentrations of resistin. As shown in Fig. 1A and B, resistin induced Transwell and wound healing migration of chondrosarcoma cells in a dose-dependent manner. However, resistin did not affect the cell viability in human chondrosarcoma cells (Supplemental Fig.S1). Previous studies have reported significant overexpression of MMP-2 in human chondrosarcoma cells [15]. Here, we found that resistin increased the protein and mRNA expressions of MMP-2, as measured by western blot, zymography assay, enzyme-linked immunosorbent assay (ELISA), and real-time quantitative polymerase chain reaction (RT-qPCR) (Fig. 1C-E). Pre-treatment of cells with an MMP-2 inhibitor or transfection with MMP-2-specific siRNA abolished resistin-induced cell migration (Fig. 1F). These results indicate that resistin promotes cell migration by up-regulation of MMP-2 expression in chondrosarcoma cells.

### The AMPK/p38 signaling pathway is involved in resistin-induced MMP-2 expression and cell migration

**Figure 2:**
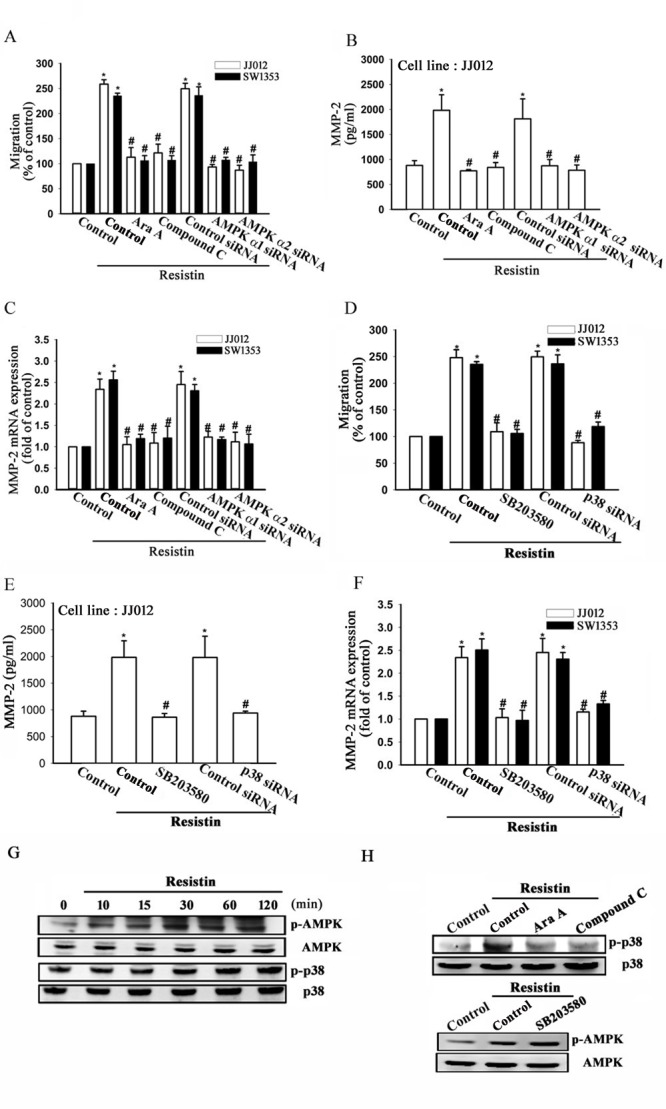
AMP-activated protein kinase (AMPK) is involved in resistin-induced matrix metalloproteinase (MMP-2) expression and cell migration JJ012 and SW1353 cells were pre-treated with Ara A (0.5 mM) and compound C (10 μM) for 30 min or pre-transfected with control, AMPKα1, or AMPKα2 siRNA for 24 h, and subsequently stimulated with resistin (3 ng/ml) for 24 h. *In vitro* migration, MMP-2 protein expression (JJ012 cells), and MMP-2 mRNA expression were measured by (A) Transwell assays, (B) enzyme-linked immunosorbent assay (ELISA), and (C) real-time quantitative polymerase chain reaction (RT-qPCR), respectively. Next, the JJ012 and SW1353 cells were pre-treated with SB203580 (10 μM) for 30 min or pre-transfected with control or p38 siRNA for 24 h, and subsequently stimulated with resistin (3 ng/ml) for 24 h. *In vitro* migration, MMP-2 protein expression (JJ012 cells), and MMP-2 mRNA expression were measured by (D) Transwell assays, (E) ELISA, and (F) RT-qPCR, respectively. (G) The JJ012 cells were incubated with resistin for the indicated time intervals, and the p-AMPK and p38 expression were examined by western blot. (H) The JJ012 cells were pre-treated for 30 min with Ara A and compound C, or SB203580 followed by stimulation with resistin. The p-p38 and p-AMPK expression were measured by western blot. The results are expressed as mean ± SEM. *, *P* < 0.05 compared with control. ^#^, *P* < 0.05 compared with the resistin-treated control group.

Recently, AMPK was shown to regulate cancer cell metastasis [31]. Hence, we investigated whether resistin-increased migration of chondrosarcoma cells is mediated by AMPK. Chondrosarcoma cells were treated with AMPK inhibitors (Ara A and Compound C) for 30 min or transfected with AMPK-specific siRNA, which abolished resistin-induced cell migration and MMP-2 expression (Fig. 2A-C). Subsequently, we directly measured AMPK phosphorylation in response to resistin and found that stimulation of cells with resistin led to an increase in phosphorylation of AMPK in a time-dependent manner (Fig. 2G). These data suggest that AMPK activation is involved in resistin-induced cell migration and MMP-2 expression in human chondrosarcoma.

In certain human diseases, AMPK is involved in p38 activation [32]. Therefore, we next investigated the role of p38 in mediating resistin-induced migration. The cells were treated with a p38 inhibitor (SB203580) or transfected with p38 siRNA, which resulted in abolished resistin-induced migration and MMP-2 expression (Fig. 2D-F). In addition, treatment of cells with resistin promoted the phosphorylation of p38 (Fig. 2G). Next, we examined the relationship between p38 and AMPK. Pre-treatment of cells for 30 min with Ara A or compound C was found to reduce p38 phosphorylation (Fig. 2H). In contrast, SB203580 did not have any effect on AMPK phosphorylation (Fig. 2H). Therefore, these results indicate that p38 is a downstream target of AMPK, and that AMPK/p38 is involved in resistin-mediated MMP-2 expression and cell migration.

### MiR-519d is an important factor in resistin-induced cell migration and MMP-2 expression

**Figure 3:**
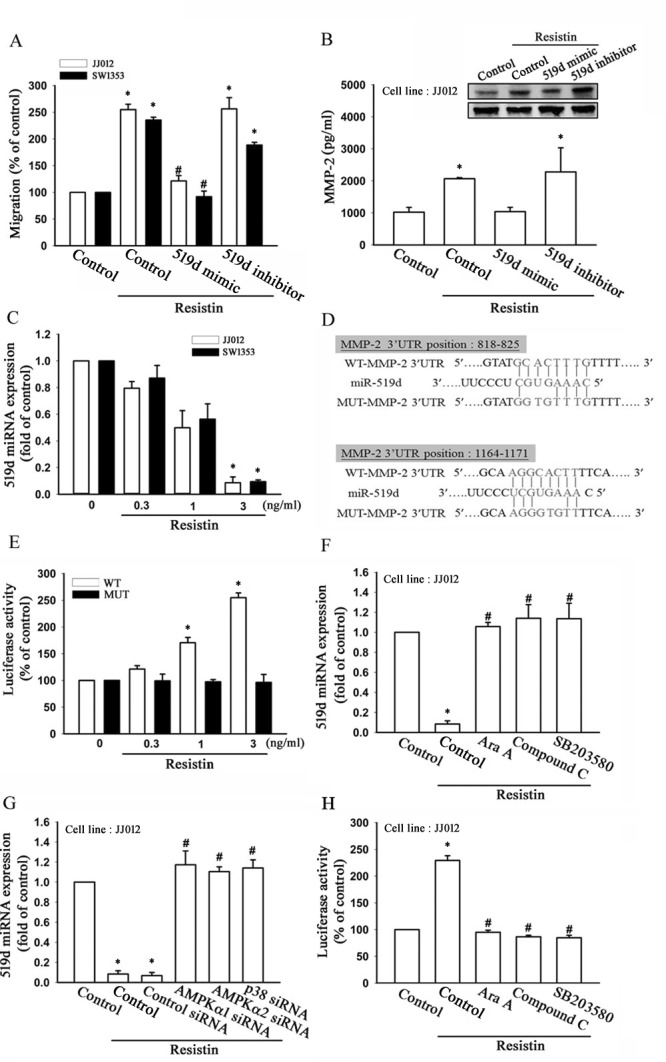
Resistin promotes cell migration and matrix metalloproteinase (MMP-2) expression by down-regulating microRNA (miR)-519d expression (A) The JJ012 and SW1353 cells were transfected with miR-519d mimic or inhibitor for 24 h, and cell migration ability was examined by Transwell assay. (B) The JJ012 cells were transfected with an miR-519d mimic or inhibitor for 24 h, and MMP-2 expression was examined by western blot (upper panel), and enzyme-linked immunosorbent assay (lower panel). (C) The JJ012 and SW1353 cells were incubated with resistin (0.3–3 ng/ml) for 24 h, and miR-519d expression was detected by real-time quantitative polymerase chain reaction. (D) Sequences of miR-519d and the potential miR-519d binding site at the MMP-2 3′ untranslated region (3′ UTR; WT-MMP-2 3′ UTR). Also shown are the nucleotides mutated in the MMP-2 3′UTR mutant (MUT-MMP2 3′ UTR). (E) The JJ012 and SW1353 cells were transfected with a wild-type or mutant MMP-2 3′ UTR luciferase plasmid for 24 h followed by stimulation with resistin (0.3-3 ng/ml) for 24 h, and the relative luciferase activity was measured. The JJ012 cells were pre-treated with AMPK and p38 inhibitors for 30 min or pre-transfected with specific siRNAs for 24 h followed by stimulation with resistin (3 ng/ml) for 24 h; and the (F, G) miR-519d expression and (H) MMP-2 3′ UTR activity were examined. The results are expressed as mean ± SEM. *, *P* < 0.05 compared with control. ^#^, *P* < 0.05 compared with the resistin-treated control group.

MiRNAs have been reported as an important regulator in cancer progression and metastasis [33], and our results indicate that resistin promotes cell migration by up-regulation of MMP-2 expression. Therefore, we next searched for possible miRNAs responsible for regulating MMP-2 expression using bioinformatic screening analyses of various databases. By overlapping the results of the DIANA-mT, miRanda, miRDB, and Targetscan databases, we found that MMP-2 was predicted to be a putative target of miR-519d. To determine whether miR-519d was indeed involved in resistin-mediated cell migration and MMP-2 expression, an miR-519d mimic or inhibitor was transfected into chondrosarcoma cells. Following resistin treatment, we found that the miR-519d mimic but not the inhibitor abolished resistin-induced cell migration and MMP-2 expression (Fig. 3A and B). By using RT-qPCR, we moreover found that resistin directly reduced miR-519d expression in a concentration-dependent manner (Fig. 3C). To demonstrate whether miR-519d specifically targeted the MMP-2 3′ UTR, we constructed luciferase reporter vectors harboring wild-type 3′ UTR of the MMP-2 mRNA (WT-MMP-2-3′ UTR) and mismatches in the predicted miR-519d binding site (MUT-MMP-2-3′ UTR; Fig. 3D). These vectors were then transfected into JJ012 and SW1353 cells after treatment with various concentrations of resistin. As shown in Fig. 3E, resistin increased luciferase activity in the WT-MMP-2-3′ UTR plasmid but not in the MUT-MMP-2-3′ UTR, indicating that miR-519d directly represses MMP-2 protein expression via binding to the 3′ UTR of human MMP-2.

To further examine whether the AMPK/p38 signaling pathway is involved in resistin-reduced miR-519d expression, we pre-treated cells with AMPK and p38 inhibitors or transfected them with AMPK and p38 siRNA, which was found to rescue resistin-inhibited miR-519d expression (Fig. 3F&G). In addition, AMPK and p38 inhibitors also reversed resistin-increased MMP-2-3′ UTR luciferase activity (Fig. 3H). Thus, resistin appears to suppress miR-519d expression through the AMPK/p38 pathway. Taken together, these data indicate that resistin promotes cell migration and MMP-2 expression by down-regulation of miR-519d through the AMPK/p38 signaling pathway.

### Resistin promotes tumor metastasis in a mouse model

**Figure 4:**
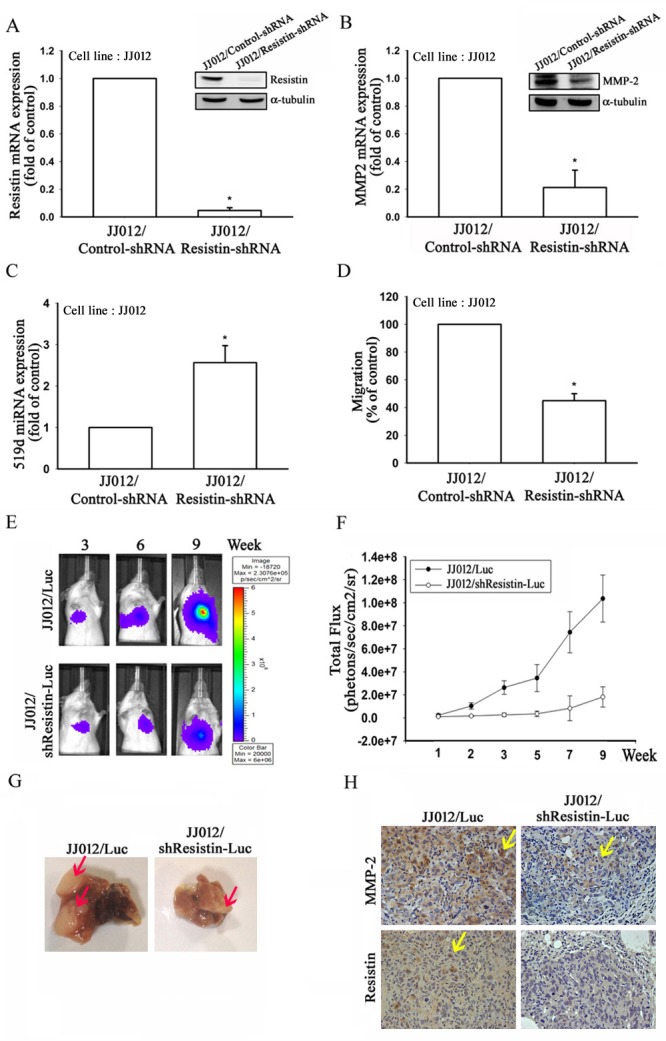
Knockdown of resistin suppresses lung metastasis *in vivo* JJ012 cells stably expressing control- or resistin-shRNA were established. The protein and mRNA expressions of (A) resistin and (B) MMP-2 were examined by western blot and real-time quantitative polymerase chain reaction (RT-qPCR), respectively. (C) MiR-519d expression in the JJ012/Control-shRNA and JJ012/Resistin-shRNA cells was detected by RT-qPCR. (D) Cell migration in the JJ012/Control-shRNA and JJ012/Resistin-shRNA cells was examined by transwell assays. (E) JJ012/Luc or JJ012/shResistin-Luc cells were injected into the lateral tail vein of severe combined immune deficient mice, and the development of lung metastasis was monitored by bioluminescence imaging at the indicated time intervals. (F) Quantification of *in vivo* bioluminescence imaging images (photons/s of lung region). (G) After 9 weeks, the mice were sacrificed, and the lung tissues were excised and photographed. (H) Immunohistochemistry of resistin and MMP-2 protein expressions in metastatic chondrosarcoma cells from the lung. The results are expressed as mean ± SEM. *, *P* < 0.05 compared with the JJ012/Control-shRNA group.

To further confirm resistin-mediated cell migration, MMP-2, and miR-519d expression *in vivo*, JJ012 cells stably expressing resistin shRNA were established. As shown in Fig. 4A, the protein and mRNA expressions were significant inhibited in stably expressing resistin shRNA cells (JJ012/Resistin-shRNA) compared to in the control cells. Furthermore, we also found that the migration ability and MMP-2 expression were dramatically decreased, and that the miR-519d expression was increased in JJ012/Resistin-shRNA cells (Fig. 4B-D).

To understand the effects of knockdown of resistin in lung metastasis *in vivo*, we established luciferase-expressing JJ012/Luc and JJ012/shResistin-Luc cells. The cells were intravenously injected into severe combined immune deficient (SCID) mice, and tumor metastasis was monitored by bioluminescence imaging. Knockdown of resistin was found to significantly suppress lung metastasis during the time course (9 weeks; Fig. 4E and F). The mice were sacrificed 9 weeks post-injection, and *ex vivo* imaging of the lungs derived from the mice showed a larger metastatic nodules in the JJ012/Luc group compared to in the JJ012/shResistin-Luc group (Fig. 4G). In addition, immunohistochemical staining showed that the protein expression of MMP-2 and resistin were significantly decreased in the JJ012/shResistin-Luc group (Fig. 4H). These results indicate that inhibition of resistin suppress lung metastasis *in vivo*.

### Up-regulation of resistin is associated with aggressive clinicopathological features of chondrosarcoma

**Figure 5:**
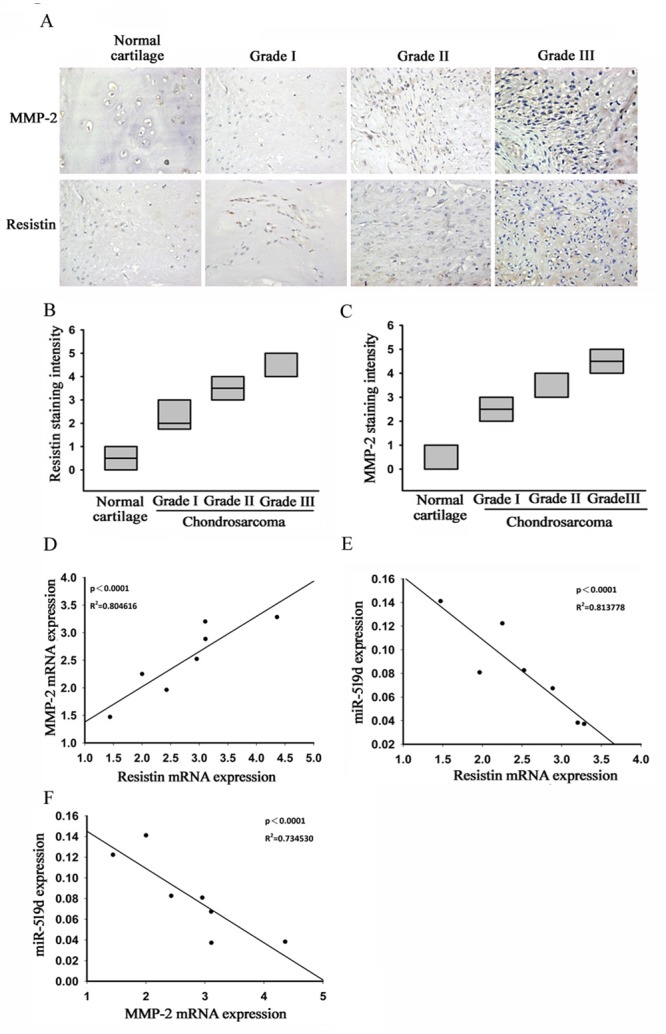
Clinical importance of resistin, matrix metalloproteinase (MMP-2), and microRNA (miR)-519d in chondrosarcoma (A-C) Immunohistochemical staining of resistin and MMP-2 in normal cartilage and chondrosarcoma tissue. Correlations between (D) resistin/MMP-2, (E) resistin/miR-519d, and (F) MMP-2/mi-519d in human chondrosarcoma tissues.

The expression levels of resistin and MMP-2 in chondrosarcoma and normal cartilage were detected by immunohistochemical staining. The protein expressions of resistin and MMP-2 in chondrosarcoma patients were found to be significantly higher than in normal cartilage (Fig. 5A-C). RT-qPCR analysis of resistin, MMP-2, and miR-519d performed in chondrosarcoma and normal samples showed a positive correlation between resistin and MMP-2; and negative correlations between resistin and miR-519d, MMP-2 and miR-519d (Fig. 5D-F).

Table I summarizes the associations of resistin expression and various clinicopathological parameters of chondrosarcoma tissues. We found that high expression of resistin was significantly associated with tumor status and metastasis. Taken together, high expressions of resistin and MMP-2, as well as reduced expression of miR-519d, were found to be associated with chondrosarcoma development and metastasis.

**Table 1:** Association of resistin expression with clinicopathological features of chondrosarcoma

Characteristic	Resistin low (n=16)	Resistin high (n=22)	*p*-value
Gender			
Female	9	12	0.4585
Male	7	10	
Stage			
IB	5	7	0.1272
IIB	7	3	
IVA	3	6	
IVB	1	6	
Tumor status			
T1	12	10	0.0343*
T2-T3	4	12	
Distant metastasis			
M0	15	16	0.0494*
M1	1	6	

* *p* <0.05

## DISCUSSION

Chondrosarcoma is the second most common primary malignancy of bone after osteosarcoma [1], and is associated with a high potential for local invasion and distant metastasis, especially to the lungs [3]. In chondrosarcoma, metastasis is the main cause of death, and a clear understanding of the metastasis biology in chondrosarcoma is hence necessary. This study characterized the effects of resistin on MMP-2 expression in human chondrosarcoma, which is known to be responsible for subsequent increased migration and metastasis. Our results indicated that resistin induced up-regulation of MMP-2 expression and tumor metastasis by down-regulation of miR-519d through the AMPK/p38 pathway.

Resistin is a recently discovered adipocytokine, which has been reported to have potent biomarker potential [34]. It is a cysteine-rich protein found in the inflammatory zone. Previous studies have shown that resistin expression is increased in various chronic inflammatory conditions such as rheumatoid arthritis, chronic kidney diseases, diabetic retinopathy, coronary heart diseases, and periodontitis [35]. Resistin has also been found to be gradually increased during the progression of certain malignancies, including breast, prostate, colon, gastric, and endometrial cancers [6, 7], and is considered a metastasis-related molecule [36]. Expression of resistin in breast cancer tissue has been considered as a marker of prognosis and hormone therapy stratification [9]; and resistin has also been demonstrated to induce adhesion of hepatocellular carcinoma cells to the endothelium [37]. However, the effects of resistin on chondrosarcoma migration are still not well recognized. Herein, we found that the expression of resistin was higher in chondrosarcoma biopsy samples than in normal cartilage. Moreover, resistin expression was found to be associated with clinical stage and metastasis, and stimulation of chondrosarcoma cells with resistin increased cell migration in a dose-dependent manner. On the other hand, inhibition of resistin expression resulted in suppression of lung metastasis *in vivo.* Accordingly, our results demonstrated that resistin promotes chondrosarcoma metastasis *in vitro* and *in vivo*.

Metastasis is a complex multi-step event leading to the formation of new tumoral sites arising from a primary tumor, and is initiated by tumor cells from the primary site invading the extracellular matrix via extracellular matrix degradation [2]. MMPs are key proteinases involved in these processes [38]. MMP-2 and MMP-9 are the unique types of proteinase that hydrolyze the bone structure of excellular matrix (type IV collagen). Thereby, they are particularly correlated with tumor metastasis [39-41]. Here, we found that resistin increased MMP-2 expression, however, resistin only slightly enhanced MMP-9 production (data not shown). In addition, MMP-2 inhibitor and siRNA abolished resistin-promoted migration and metastasis. Therefore, MMP-2 plays an important role than MMP-9 in resistin-mediated cell motility.

AMPK expression has been found to correlate with various cancers, including ovarian, hepatocellular, pancreatic, breast, and gallbladder cancers [17-22], and a previous study found that the tumors in Peutz-Jeghers syndrome may result from deficient activation of AMPK as a consequence of inactivation of serine/threonine kinase 11, the major upstream kinase required for AMPK activation [42]. Activation of AMPK moreover results in cell cycle arrest mediated via the p53 tumor suppressor protein [43]. Therefore, the AMPK pathway is thought to play a role in tumor suppression. Recently, several studies have shown that AMPK also plays an important role in metastasis through its effects on cell migration; and it has also been found that AMPK stimulates cell motility via microtubule polymerization, and that silencing AMPK expression results in disrupted front-rear polarity, as well as directional migration defects [44-46]. Our results indicated that resistin induced cell migration by activation of AMPK phosphorylation in human chondrosarcoma cells. AMPK is believed to play an important role in metastasis, and may hence represent a potential target for the development of new anticancer drugs, in particular those targeting metastasis.

In conclusion, metastasis plays a critical role in the progression of tumors and is the main cause of all cancer-related deaths. Today, chondrosarcoma is resistant to both chemotherapy and radiation, and there is no specific standardized therapy that has been proven to be effective for chondrosarcoma [3]. In this study, we found that increased resistin expression strongly stimulates chondrosarcoma cell migration and metastasis. Resistin appears to promote cell migration and MMP-2 expression through inhibition of miR-519d via the AMPK/p38 signaling pathway. Therefore, targeting resistin or its signaling pathways may enable the development of novel molecular targeted treatments for chondrosarcoma.

## Materials and Methods

### Materials

Anti-rabbit and anti-mouse IgG-conjugated horseradish peroxidase; rabbit polyclonal antibodies specific for α-tubulin, AMPK, phospho(p)-AMPK (Thr^172^), p38, p-p38; mouse monoclonal antibodies specific for resistin and MMP-2; and AMPKα1/2 siRNA were purchased from Santa Cruz Biotechnology (Santa Cruz, CA). ON-TARGETplus MMP2, p38, and control siRNAs were purchased from Dharmacon Research (Lafayette, CO). AMPK inhibitors (Ara A and compound C), p38 inhibitor (SB203580), MMP-2 inhibitor, and human MMP-2 ELISA kits were purchased from Calbiochem (San Diego, CA). Recombinant human resistin was purchased from PeproTech (Rocky Hill, NJ). MiR-519d mimic and inhibitor were purchased from Invitrogen (Carlsbad, CA). All other chemicals were purchased from Sigma-Aldrich (St. Louis, MO).

### Cell culture

The human chondrosarcoma cell line JJ012 was donated by the laboratory of Dr. Sean P. Scully (University of Miami School of Medicine; Miami, FL) [47], and the cells were cultured in Dulbecco's Modified Eagle's Medium (DMEM)/α-MEM supplemented with 10% fetal bovine serum (FBS). The human chondrosarcoma cell line SW1353 was obtained from the American Type Culture Collection, cultured in DMEM supplemented with 10% FBS, and maintained at 37°C in humidified 5% CO_2_ atmosphere.

### Patients and specimen preparation

The study protocol was approved by the Institutional Review Board (No. DMR 98-IRB-274) of China Medical University Hospital, and all subjects gave informed written consent before enrollment. The specimens of normal cartilage or tumor tissue were obtained from patients who were had been diagnosed with osteoarthritis or chondrosarcoma and had undergone surgical resection at China Medical University Hospital. The histologic grades (on a scale of I to III) of each chondrosarcoma patients were according to the World Health Organization (WHO) Classification of Tumours of Soft Tissue and Bone (2013) [48]. The tissue samples for miRNA or mRNA examination were sharply excised, placed in sterile tubes, frozen immediately in liquid nitrogen, and stored at −80°C until analysis.

### Migration assay

The migration assay was performed using transwell plates (Costar, NY; pore size, 8 mm). Approximately 1×10^4^ cells in 100 ml of serum-free medium were placed in the upper chamber, and 300 ml of the same medium was placed in the lower chamber. The plates were incubated for 24 h at 37°C in 5% CO_2_, and the cells were then fixed in 3.7% formaldehyde for 15 min and stained with 0.05% crystal violet in phosphate buffered saline (PBS) for 15 min. Cells on the upper side of the filters were removed with cotton-tipped swabs, and the filters were washed with PBS. Cells on the underside of the filters were examined and counted under a microscope. Each clone was plated in triplicate for each experiment, and each experiment was repeated at least three times.

### Immunohistochemical staining

Human chondrosarcoma tissue sections were deparaffinized with xylene and rehydrated through addition of ethanol at decreasing concentrations. Endogenous peroxidase activity was blocked with 3% hydrogen peroxide in methanol for 10 min. Heat-induced antigen retrieval was carried out for all sections in 0.01 M sodium citrate buffer, pH 6 at 95°C for 25 min. Human resistin and MMP-2 antibodies were applied at a dilution of 1:150 and incubated at 4°C overnight. The antibody-binding signal was detected using the NovoLink Polymer Detection System (Leica Microsystems) and visualized using 3-3′-diaminobenzidine. The sections were counterstained with hematoxylin. The immunohistochemistry results were scored by taking into account the percentage of positive detection and the intensity of the staining.

### ELISA

Human chondrosarcoma cells were cultured in 24-well plates. After reaching confluence, the cells were changed to a serum-free medium. Subsequently, the cells were treated with resistin alone for 24 h, or pre-treated with pharmacological inhibitors or transfected with specific siRNAs, followed by stimulation with resistin for 24 h. After treatment, the medium was removed and stored at −80°C, and the MMP-2 expression in the medium was determined using the MMP-2 ELISA kit according to the manufacturer's protocol.

### Luciferase activity assay

The 3′ UTRs of the human MMP-2 gene were amplified by PCR. The 3′ UTRs were cloned in the pGL2-Control vector (Promega, Madison, WI, USA), downstream of the reporter gene. The predicted MMP-2 binding site, identified by the miRDB (http://mirdb.org/miRDB), were amplified by PCR and cloned in the pGL2-Control vector, upstream of the reporter gene. Mutant plasmids were generated using a QuikChange Site-Directed Mutagenesis kit (Stratagene, Cedar Creek, TX, USA).

### Western blot analysis

Protein concentration was determined using the Thermo Scientific Pierce BCA Protein Assay Kit (Thermo Fisher Scientific Inc., USA). Proteins were resolved on odium dodecyl sulfate polyacrylamide gel electrophoresis and transferred to Immobilon^®^ polyvinyl difluoride membranes. The blots were blocked with 4% bovine serum albumin for 1 h at room temperature, and incubated with the primary antibodies for 1 h at room temperature. After 3 washes in Tris-buffered saline with 0.05% Tween 20, the blots were subsequently incubated with a donkey anti-rabbit or anti-mouse peroxidase-conjugated secondary antibody for 1 h at room temperature. The blots were visualized by enhanced chemiluminescence using Kodak X-OMAT LS film (Eastman Kodak, Rochester, NY). Quantitative data were obtained using a computing densitometer and ImageQuant software (Molecular Dynamics, Sunnyvale, CA).

### Real-time quantitative PCR

Total RNA was extracted from chondrosarcoma cells using a TRIzol kit (MD Bio Inc., Taipei, Taiwan). The reverse transcription reaction was performed using 2 μg of total RNA that was reverse transcribed into cDNA using an oligo(dT) primer. RT-qPCR analysis was carried out using TaqMan^®^ one-step PCR Master Mix (Applied Biosystems, Foster City, CA, USA). Total complementary DNA (100 ng/25 μL reaction) was mixed with sequence-specific primers and TaqMan^®^ probes according to the manufacturer's instructions. Sequences for all target gene primers and probes were purchased commercially (β-actin was used as the internal control) (Applied Biosystems). qPCR assays were carried out in triplicate using a StepOnePlus sequence detection system. The cycling conditions were 10 min of polymerase activation at 95°C, followed by 40 cycles at 95°C for 15 s and 60°C for 60 s.

For miRNA detection, reverse transcription was performed using Mir-X™ miRNA First-Strand Synthesis and SYBR^®^ RT-qPCR. U6 snRNA was used for normalization. The specific forward primer of miR-519d was as follows: 5′-CAAAGTGCCTCCCTTTAGAGTG-3′. The threshold was set above the non-template control background and within the linear phase of target gene amplification to calculate the cycle number at which the transcript was detected (denoted as CT).

### *In vivo* metastasis model

The cells (JJ012/Luc or JJ012/shResistin-Luc) were washed and resuspended in PBS. Subsequently, a unit suspension containing 5×10^6^ cells in 100 μl PBS was injected into the lateral tail vein of 5-week-old SCID mice. Lung metastasis was monitored using an *in vivo* imaging system (Xenogen IVIS imaging system). After 9 weeks, the mice were humanely sacrificed by an overdose of anesthetics. Subsequently, the lungs were removed and photographed. The metastatic chondrosarcoma cells were excised from the lungs and fixed in 10% formalin, embedded in paraffin, and processed for immunohistochemical staining with resistin and MMP-2.

### Statistical analysis

All data are expressed as mean ± standard error. The differences between groups were analyzed using the Student's *t*-test or the χ^2^ test for homogeneity. The differences were considered significant if the *p* value was less than 0.05.

## SUPPLEMENTARY MATERIAL, FIGURE


