# Correction: Resistin promotes tumor metastasis by down-regulation of miR-519d through the AMPK/p38 signaling pathway in human chondrosarcoma cells

**DOI:** 10.18632/oncotarget.26303

**Published:** 2018-10-30

**Authors:** Chun-Hao Tsai, Hsiao-Chi Tsai, Ho-Ning Huang, Chih-Hung Hung, Chin-Jung Hsu, Yi-Chin Fong, Horng-Chaung Hsu, Yuan-Li Huang, Chih-Hsin Tang

**Affiliations:** ^1^ Department of Medicine and Graduate Institute of Clinical Medical Science, China Medical University, Taichung, Taiwan; ^2^ Department of Orthopedic Surgery, China Medical University Hospital, Taichung, Taiwan; ^3^ Graduate Institute of Basic Medical Science, China Medical University, Taichung, Taiwan; ^4^ Department of Biotechnology, College of Health Science, Asia University, Taichung, Taiwan; ^5^ School of Chinese Medicine, College of Chinese Medicine, China Medical University, Taichung, Taiwan; ^6^ Department of Pharmacology, School of Medicine, China Medical University, Taichung, Taiwan

**This article has been corrected:** Due to an accidental duplication, the images for Grade I MMP-2 staining and Normal Cartilage Resistin staining in Figure 5A are identical. The corrected Figure 5A is shown below. The authors declare that these corrections do not change the results or conclusions of this paper.

**Figure 5:**
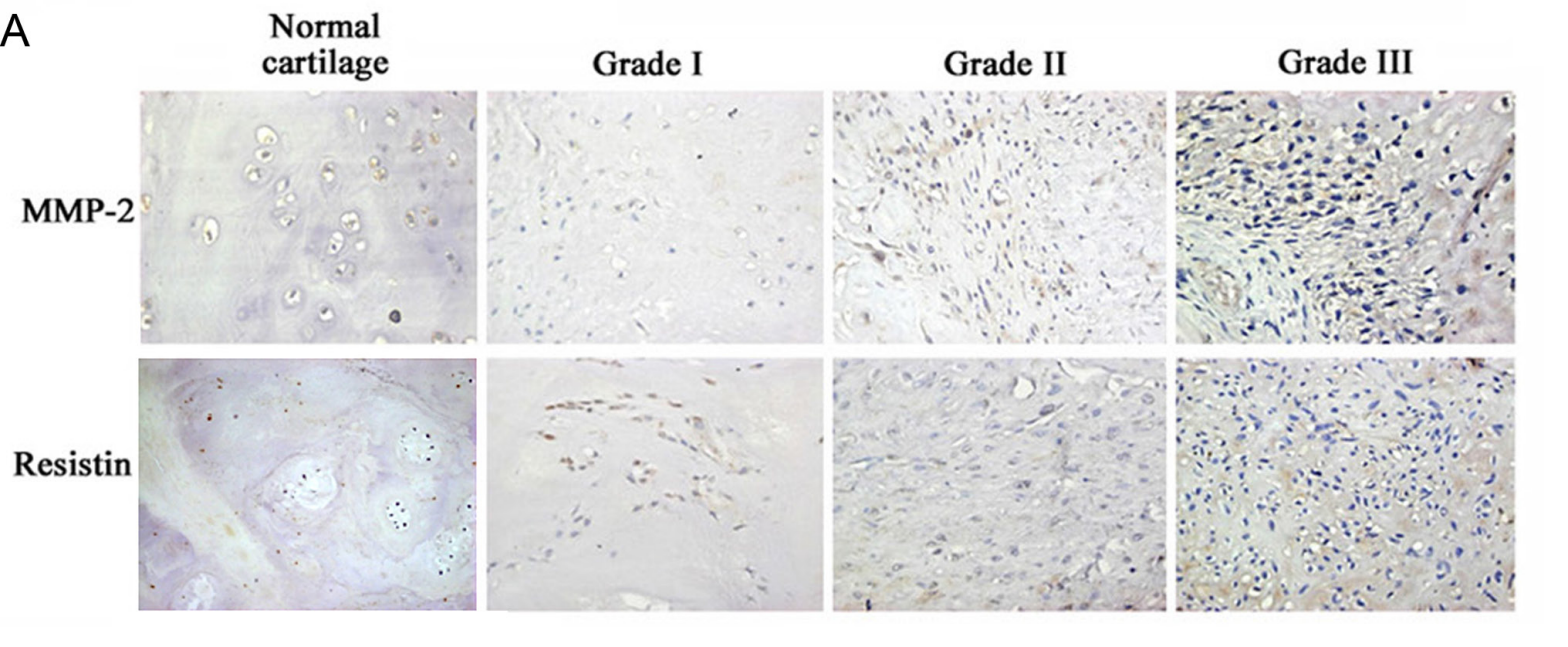
Clinical importance of resistin, matrix metalloproteinase (MMP-2), and microRNA (miR)-519d in chondrosarcoma. (A-C) Immunohistochemical staining of resistin and MMP-2 in normal cartilage and chondrosarcoma tissue. Correlations between (D) resistin/MMP-2, (E) resistin/miR-519d, and (F) MMP-2/mi-519d in human chondrosarcoma tissues.

Original article: Oncotarget. 2015; 6:258-270. https://doi.org/10.18632/oncotarget.2724

