# Supplementary material for: Resistin promotes tumor metastasis by down-regulation of miR-519d through the AMPK/p38 signaling pathway in human chondrosarcoma cells
Source: Oncotarget. 2014 Nov 6;6(1):258–70. doi: 10.18632/oncotarget.2724 (PMC4381593; doi:10.18632/oncotarget.2724)
Supplement: Supplementary file 1 [file oncotarget-06-258-s001.pdf]

Resistin promotes tumor metastasis by down-regulation of miR-519d through the AMPK/p38 signaling pathway in human chondrosarcoma cells

Supplementary Material

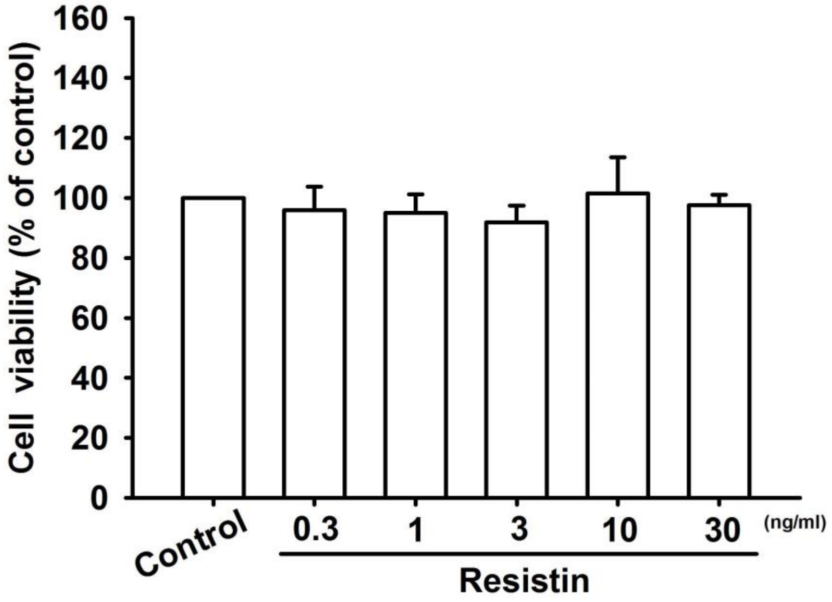

Figure S1: Resistin did not affect the cell viability in human chondrosarcoma cells. JJ012 cells were incubated with resistin (0.3–30 ng/ml) for 24 h. The cell viability was analyzed by using the MTT assay. The results are expressed as mean ± SEM.
